# Advances in Mesenchymal Stem Cell Therapy for Osteoarthritis: From Preclinical and Clinical Perspectives

**DOI:** 10.3390/bioengineering10020195

**Published:** 2023-02-02

**Authors:** Zehui Lv, Xuejie Cai, Yixin Bian, Zhanqi Wei, Wei Zhu, Xiuli Zhao, Xisheng Weng

**Affiliations:** 1 Department of Orthopaedics, Peking Union Medical College Hospital, Chinese Academy of Medical Sciences & Peking Union Medical College, Beijing 100730, China; 2 Department of Medical Genetics, Institute of Basic Medical Sciences, School of Basic Medicine, Chinese Academy of Medical Sciences & Peking Union Medical College, Beijing 100005, China; 3 Department of State Key Laboratory of Complex Severe and Rare Diseases, Peking Union Medical College Hospital, Chinese Academy of Medical Sciences & Peking Union Medical College, Beijing 100730, China

**Keywords:** osteoarthritis, mesenchymal stem cells, preclinical trials, clinical trials, cartilage regeneration

## Abstract

The prevalence of osteoarthritis (OA), a degenerative disorder of joints, has substantially increased in recent years. Its key pathogenic hallmarks include articular cartilage destruction, synovium inflammation, and bone remodeling. However, treatment outcomes are unsatisfactory. Until recently, common therapy methods, such as analgesic and anti-inflammatory treatments, were aimed to treat symptoms that cannot be radically cured. Mesenchymal stem cells (MSCs), i.e., mesoderm non-hematopoietic cells separated from bone marrow, adipose tissue, umbilical cord blood, etc., have been intensively explored as an emerging technique for the treatment of OA over the last few decades. According to existing research, MSCs may limit cartilage degradation in OA by interfering with cellular immunity and secreting a number of active chemicals. This study aimed to examine the potential mechanism of MSCs in the treatment of OA and conduct a thorough review of both preclinical and clinical data.

## 1. Introduction

**Figure 1:**
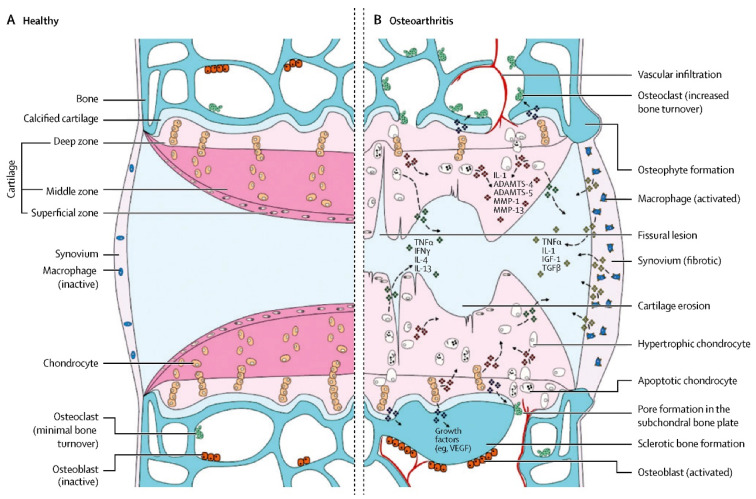
Comparison between normal joints and osteoarthritis. The roles of mechanical, metabolic, and inflammatory factors are identified. Reprinted from [2].

Ten percent to fifteen percent of the global population suffers from osteoarthritis (OA), a condition characterized by cartilage deterioration and synovitis. It is a rapidly developing modern disease [1]. Figure 1 summarizes the pathological process in detail [2]. There are many risk factors, such as sex, age, trauma, obesity and genetics. Since the middle of the last century, OA has not only shown its highest worldwide incidence rate [3] but has also forced various developed countries to bear heavy economic burdens because it is a crippling disease [4,5]. Currently, there is no ideal specific drug used for the clinical treatment of OA. To relieve inflammation and discomfort in early stages, nonsteroidal anti-inflammatory medications and analgesics, such as glucosamine, chondroitin supplements, and intra-articular local injections of corticosteroids are primarily employed [6]. Though it is an intrusive procedure that is frequently accompanied by significant uncontrolled complications, joint replacement plays a prominent role in advanced cases [7,8]. However, the service life of the prosthesis and the functional recovery of the damaged limbs are not as positive as we originally anticipated and can even be described as restricted [6]. As a result, research into a more secure and efficient treatment for OA is crucial.

In recent years, several academics have conducted extensive research on MSCs, which are specialized adult stem cells used in many studies [9] due to their capacity for self-renewal and differentiation. MSCs are also widespread throughout the body, suggesting that they might be used as an alternative cell source in OA therapy. Studies have indicated that the surface layer of cartilage sustains the vast majority of damage during OA [10,11]. Importantly, several MSC types have been implicated in the differentiation of chondrocytes. In the process of cartilage remodeling, the surface cartilage is minimally impacted; moreover, following remodeling, bidirectional mitotically active cells can horizontally or vertically replenish chondrocytes [12,13].

In the study of MSCs transplanted into articular cavities in arthritis models, e.g., for mice [14] and horses [15], considerable regeneration and repair in articular cartilage have been observed; moreover, there is evidence that MSCs reduce cartilage lesions by restraining the onset of inflammation. MSCs were found to limit the breakdown of proteoglycan in the cartilage of a rabbit arthritis model by decreasing the production of tumor necrosis factor (TNF)-α and MMP-1 [16]. In addition, by releasing growth factors (such as TGF-beta and IL-6) and antioxidant compounds, MSCs conspicuously suppress apoptosis and fibrosis [17,18]. The aim of this review was to explore the probable mechanism of MSCs in the therapeutic treatment of OA based on the current state of research.

## 2. Characteristics of MSCs for the OA Therapy

**Figure 2:**
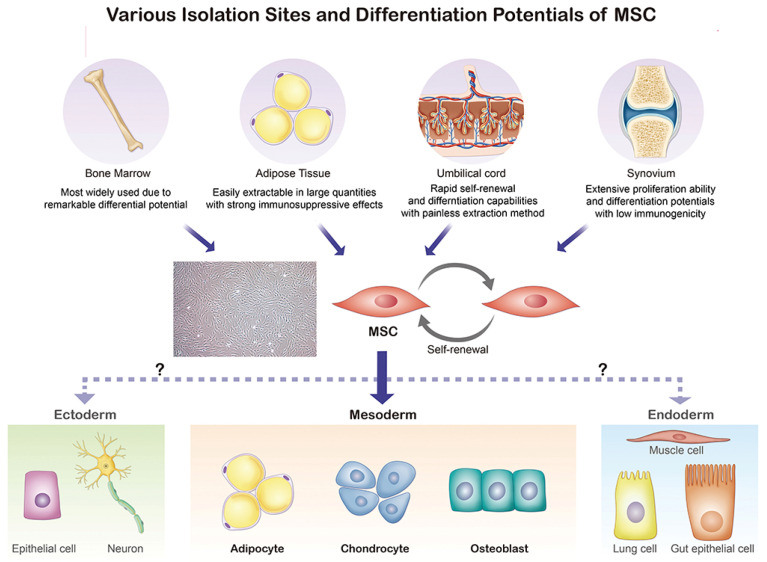
Origin and differentiation directions of MSCs. Reprinted from [24]. MSC, mesenchymal stem cell.

MSCs, as specific types of adult stem cells, possess great potential in bone tissue engineering and regenerative therapy due to their capacity for self-renewal and differentiation [19,20,21]. The multipotency, wide availability, and low immunogenicity of MSCs have made them a hot topic in the bioremediation field. In 1976, Friedenstein et al. [22] identified and prepared MSCs from different tissues, including fat, placenta, umbilical cord, synovium, bone, and dental pulp [23]. As shown in Figure 2, MSCs, which are mainly extracted from bone marrow, adipose tissue, umbilical cord, and the synovium, possess the ability to differentiate into adipocytes, chondrocytes, and osteoblasts.

The International Society for Cell Therapy established three criteria for defining MSCs. First and foremost, they exhibit adhesion growth characteristics under conventional culture conditions. Secondly, MSCs attached to culture plastic must display significant levels of CD105, CD73, and CD90, but not CD45, CD34, CD14, CD11b, CD79a, CD19, and HLADR surface markers after being detected with flow cytometry. Thirdly, MSCs can differentiate into bone-forming cells (chondrocytes and osteoblasts) or fat-storing cells (adipocytes) when cultivated in a certain induction media [25].

In general, the proliferative capacity of MSCs obtained from fetal tissue is more outstanding than that of MSCs obtained from adult tissue; additionally, umbilical cord, amniotic membrane, and fat sources are more accessible and possess a higher proliferative capacity, while the proliferation ability of bone marrow-derived MSCs (BM-MSCs) is relatively inadequate [26,27]. MSCs isolated from adipose tissue are the most genetically and morphologically stable, and they proliferate best over an extended period of incubation [28]. Gene expression analysis revealed that BM-MSCs express higher levels of osteogenic differentiation-related genes than MSCs isolated from umbilical cords [29,30]. Nevertheless, compared with BM-MSCs, umbilical cords MSCs can produce more cell growth factors with a lower immunogenic potential and better immunomodulatory functions. Studies have demonstrated that MSCs extracted from the amniotic membrane and fat are superior to those collected from bone marrow, and MSCs sourced from umbilical cord blood show the weakest immunosuppressive potential [29,30].

Adipose tissue-derived MSCs (AD-MSCs) demonstrated worse cell morphology and matrix formation than BM-MSCs during in vitro chondrogenesis, whereas their adipogenic differentiation capacity was found to be comparable [31]. Although it is difficult to discriminate between the chondrogenic differentiation of BM-MSCs and AD-MSCs in monolayers, only the chondrogenic capacity of BM-MSCs was shown to be enhanced in three-dimensional cell culture [32]. When compared with the same donor’s subcutaneous adipose tissue, AD-MSCs in the subpatellar fat of an osteoarthritis knee manifested an enhancement in chondrogenesis and osteogenesis [33]. However, cell yields from inhaled tissues vary between BM-MSCs and AD-MSCs, with the latter having a higher survival rate than the former [31]. Only 0.001–0.01% of the 6 × 10^6^ nucleated cells that may be generated from a milliliter of bone marrow extract are MSCs [34]. On the other hand, adipose tissue (2 × 10^6^ cells per gram) contains roughly 10% bone marrow MSCs [35]. Due to the absence of clinical research comparing the performance of MSCs derived from diverse sources, it is unclear which cells perform the best for OA healing. Intriguingly, OA was found to be alleviated by implanting a tiny adipose tissue depot obtained from wild-type mice or mouse embryonic fibroblasts that had spontaneously become adipocytes [36].

The initially investigated MSCs were BM-MSCs; however, due to their high invasiveness and limited quantity in vivo, they have been increasingly supplanted by MSCs from other sources, the most striking of which are human umbilical-cord-derived MSCs (HUC-MSCs). Thanks to their robust in vitro proliferation capacity, minimal immunogenicity, ease of isolation and culture, and sustained multidirectional differentiation potential, HUC-MSCs are frequently utilized. HUC-MSCs suppress an inflammatory response caused by IL-1 and repair-impaired cartilage by differentiating into cartilage [37]. Moreover, human-induced pluripotent stem-cell-derived MSCs (iPSC-MSCs) generated from human-induced pluripotent stem cells are a unique form of stem cells with an enhanced regenerative capacity compared with conventional stem cells, and they are able to alter highly differentiated adult somatic cells through genetic engineering. Theoretically, all adult somatic cells could be reprogrammed into iPSC-MSCs, which exhibit a higher proliferation capability than conventional MSCs. The formation of new hyaline cartilage at the joint surface defect areas following iPSC-MSC transplantation into a New Zealand rabbit model suggested the reparative action of iPSC-MSCs [38]. Moreover, Cheng et al., discovered that iPSC-MSCs may release certain substances via the paracrine route to prevent the cleavage of caspase and contribute to the control of inflammation, indicating a possible function for iPSC-MSCs in immunosuppression [39].

## 3. Underlying Treating Mechanism of MSCs for OA

The therapeutic potential of MSCs in terms of immunological and inflammatory disorders has been explored in several clinical studies [40,41]. BM-MSCs produce substances that are immunoregulatory and anti-inflammatory [42]. Therefore, MSCs are able to efficiently downregulate immune inflammatory processes and boost tissue regeneration because they display particular immunological traits and activities. When tissues are damaged, local tissue progenitor cells that raise the potential to modulate the immune system are called upon and activated [43]. OA is characterized by an influx of immune cells, mostly monocytes/macrophages and then T cells, into the synovium. Furthermore, OA synovia contain mast cells, natural killer (NK) cells, dendritic cells, B cells, and granulocytes. This subject has been more thoroughly covered elsewhere [44]. There might be three ways to accommodate the immune system. Firstly, BM-MSCs may control innate immunity by suppressing the development of mature dendritic cells [45] and lowering the cytotoxicity of NK cells [46]. Secondly, MSCs may modify acquired immunity by preventing cell death (apoptosis) and slowing the development of T and B cells [42,47]. Finally, MSCs may switch macrophages from an inflammatory (M1) phenotype to a restorative (M2) phenotype [48]. It has been demonstrated that BM-MSCs could induce a switch in macrophage phenotype from the pro-inflammatory M1 phenotype, which generates IL-1 and TGF-β, to the anti-inflammatory and chondrogenic phenotype, which produces IL-10, IL-rheumatoid arthritis, and TGF-β [49]. The impact of BM-MSCs on macrophage polarization is mediated by TNF-α, which stimulates gene/protein 6 (TSG-6), prostaglandin E2 (PGE2), and indoleamine 2-dioxygenase 3-dioxygenase (IDO) [49]. The immune switch from M1 to M2 could be beneficial in relieving OA by reducing periarticular inflammation. Moreover, BM-MSCs suppress pathogenic immune responses, remove infections, and improve local cell function [50].

However, the precise mechanism through which BM-MSCs promote joint repair is not yet known. It would be wonderful if MSCs could be implanted and immediately develop into chondrocytes [51,52,53]. Nonetheless, the survival rate of implanted MSCs was found to be low [54], and 50 days after injection, BM-MSCs could not be detected [55].

It has been shown in some studies that implanted MSCs may boost stem/progenitor cell recruitment and cartilage differentiation by secreting substances that encourage the proliferation and anabolism of articular chondrocytes [54,56,57]. Factors produced by MSCs have been shown to affect synovium and articular chondrocytes, which control anabolic and catabolic processes [58,59] and increase the synthesis of molecular mediators of inflammation and chondrogenesis [54,60,61]. Moreover, by downregulating the neuralgia pathway, reduced inflammation may alleviate neuropathic pain.

## 4. Methods for Supplying MSCs

Traditionally, the autotransplantation of MSCs was utilized to treat osteoarthritis. The majority of OA patients are elderly people whose cell capacity to proliferate and differentiate in vitro is diminished. For these patients, it is impracticable to create MSCs in a short period of time, therefore restricting the therapeutic use of autologous MSCs. It has been elucidated that, unlike other stem cells, MSCs possess unique immunosuppressive and immunological tolerance, as well as reduced immunogenicity, hence preventing allograft rejection in allotransplantation [62]. Consequently, the allotransplantation of MSCs is deemed safe, and it is expected to be extensively applied in clinical settings. Most research has employed a single injection of allogeneic MSCs to relieve OA; however, a few studies have reported that several injections may further boost therapy efficacy [63,64,65].

The need to intravenously inject MSCs to treat OA has been infrequently documented; the majority of injections are directly applied within the articular cavity. This technique promotes the healing of damaged cartilage along with the direct differentiation of MSCs into chondrocytes and the production of a matrix in the damaged region; however, other research indicates that MSCs have a limited lifespan [55]. Injecting MSCs in the form of a gel into the articular cavity was shown to improve patient outcomes [66], though the impact of implanting MSCs into cartilage defects under arthroscopy appears more favorable [67]. In an animal study, BM-MSCs were cultured and implanted with a collagen hyaluronic acid scaffold, greatly upregulating the production of II-type collagen in cartilage defects [68].

Furthermore, the quantity of MSCs may play a significant role in the recovery and prognosis of OA patients. Different donors, recipients, cell growth procedures, and transmission generations all make it challenging to standardize the appropriate dosage of MSCs. For instance, the dosages of MSCs produced from bone marrow range between 8 × 10^6^ [63] and 10 × 10^6^ [69,70], sometimes reaching more than 20 × 10^6^ [71], but they are mostly in the range of 1–100 × 10^6^ [72]. Studies have testified that the efficiency of MSCs for the purpose of treating OA rises to a certain extent with increased dosage [65].

Multiple injections and MSC doses might represent critical prognostic variables for healing OA. The ideal consumption of MSCs remains uncertain, and further studies are needed. It has been determined that injections of >1 × 10^7^ MSCs are required for effectual repair [65,73]. Infusions of BM-MSCs in numbers ranging from 8 × 10^6^ [63] or >10 × 10^6^ [70,74] to >20 × 10^6^ [64,69] have been employed, with BM-MSCs producing superior outcomes than controls except at the lowest dosage of MSCs [63,75]. Numerous injections are more suitable for an allogeneic MSC method, although they may be applied for autologous approaches as well [74].

## 5. Preclinical Trials

Intra-articular injections of MSCs have been proven to enhance joint performance in animal experiments, providing an encouraging reference for subsequent clinical trials (Table 1).

### 5.1. MSCs for the Treatment of Rat OA

Horie et al. [76] treated huge meniscus defects via the direct intra-articular injection of synovium-derived MSCs (Sy-MSCs) in mice. The results showed that stem cells could adhere to meniscus defects and directly differentiate into meniscal cells to promote meniscus repair and regeneration. A few years later, the team conducted another study centered around the articular injection of rat MSCs or human MSCs to treat a rat model of OA induced by meniscectomy. It was found that human MSC injection could increase the expression of the type II collagen of rats and inhibit the progression of osteoarthritis [77]. Cui et al. [78] injected different concentrations of BM-MSCs into a post-traumatic rat OA model and found that the Mankin score was significantly improved and the mRNA expression of type II collagen increased. However, while some studies have shown that the local injection of MSCs into articular cavities to treat OA induced by iodoacetic acid can significantly improve joint function, statistical differences in improving cartilage, subchondral bone pathology, and synovitis have been found [79].

Furthermore, Ozeki et al. [61] showed that Sy-MSCs prevented the progression of collagenase-induced osteoarthritis in a rat model. They also evaluated the number of injections of Sy-MSCs required to treat OA in their mouse model. They showed that injected Sy-MSCs increased the expression of genes associated with chondroprotection such as PRG-4 and BMP-2 by more than 50 fold. In addition to chondroprotection, they also noted the enhanced expression of TSG-6, which is responsible for immune regulation and blocking the inflammatory cascade.

In addition, He et al. [80] explored the effect of BM-MSC exosome injection on cartilage damage and pain relief in a rat OA model, and their experimental results demonstrated the significant upregulation of COL2A1 proteins and the downregulation of MMP13 proteins in cartilage tissue after exosome therapy by weakening the inhibitory effect of IL-1β on chondrocyte proliferation and migration. Xing et al. [81] injected rats with embryonic stem-cell-derived MSCs and found favorable improvements in both short-term and long-term rat OA after injection. Yang et al. [82] evaluated the effect of AD-MSCs on cartilage damage in rat OA models and found that they did affect articular cartilage regeneration.

### 5.2. MSCs for the Treatment of Rabbit OA

In a rabbit OA model, Zellner et al. [68] implanted collagen hyaluronic acid scaffolds after the expansion of bone marrow-derived mesenchymal stem cells and pressed the cell seed scaffold into cartilage defects, potentially significantly upregulating the expression and increasing the yield of type II collagen genes. In the study conducted by Mata et al. [83], dental pulp MSCs were implanted into a rabbit cartilage injury model, and obvious cartilage regeneration was observed 3 months after operation. Riester et al. [84] injected cultured and expanded human AD-MSCs into the knee joints of rabbits; tolerance was good, and no evidence of intra-articular joint tissue damage was found. Jeon et al. [85] reported that the synovial fluid and joints of rabbits treated with UCB-MSCs showed reduced inflammation and improved proteoglycan and collagen type 2 production and structure.

There have also been many experiments using Sy-MSCs. Pei et al. [86] demonstrated that regenerated cartilage appeared as smooth hyaline cartilage at a 6-month follow-up. Lee et al. [87] investigated the use of platelet-rich plasma to deliver Sy-MSCs to regenerate full-thickness cartilage lesions; their treated group showed significantly improved microscopic and macroscopic scores at 6 months of follow-up. Shimomura et al. [88] combined Sy-MSCs with hydroxyapatite (HA) and implanted full-thickness cartilage lesions in rabbits. They demonstrated that subjects using Sy-MSCs and HA exhibited faster integration and improved the appearance of osteochondral bone compared with controls using only HA, which exhibited osteoarthritic features at a 6-month follow-up. Li et al. [89] characterized the cartilage quality of rabbit knee osteochondral lesions repaired by Sy-MSCs and found that the treated animals had higher tissue quality. Schmal et al. [90] compared the ability of heterologous Sy-MSCs to repair cartilage lesions in rabbit femurs. They noted an increased macroscopic regenerative capacity in the Sy-MSC group compared with the control group.

### 5.3. MSCs for the Treatment of Goat OA

Murphy et al. [91] injected labeled BM-MSCs into goat joints to initiate cartilage tissue regeneration, but a relative lack of labeled MSCs was found in the regenerated cartilage area. Saw et al. [92] performed the intra-articular injection of a bone marrow aspiration primer combined with hyaluronic acid after a surgery-induced microfracture in a sheep model. The results showed that tissue integration and tissue repair could be improved. In the same model, after the goat OA model was operated on, allogeneic AD-MSCs cells combined with hyaluronic acid (HA) were injected into the joints of a sheep OA model. An evaluation of magnetic resonance imaging (MRI), macroscopy, microcomputer tomography, and cartilage-specific staining showed that the AD-MSC + HA treatment group retained the typical characteristics of articular cartilage, effectively blocked the progress of OA, and promoted cartilage regeneration [93].

### 5.4. MSCs for the Treatment of Horse OA

McIlwraith et al. [94] made a 1 cm^2^ cartilage defect through arthroscopy in the knee joints of horses aged 2.5 to 5.0 while the subchondral bone was treated with a microfracture. One month after operation, hyaluronic acid containing 20 × 10^6^ BM-MSCs was injected into the articular cavity of some horses and hyaluronic acid without stem cells was injected into the articular cavity of control horses. At 12 months after operation, an evaluation was conducted. There was no significant difference in clinical efficacy between the two groups.

### 5.5. MSCs for the Treatment of Dog OA

A randomized, double-blind, placebo-controlled trial of autologous AD-MSCs for chronic OA showed significant improvements in the claudication index, pain score, and range of motion in the AD-MSC group [95]. Additionally, Black et al. [96] confirmed that after a single intra-articular injection of AD-MSCs, the claudication and range of movement of dogs were significantly improved. In a recent study, Huňáková et al. [97] found that the double intra-articular administration of canine adipose tissue derived from Labrador retrievers improved the functional ability of dogs.

### 5.6. MSCs for the Treatment of Pig OA

Furthermore, in the pig OA model, BM-MSC injection once again showed preclinical effects compared with a control group, as cartilage defect healing was improved [98]. Several studies have used porcine models to evaluate porcine Sy-MSCs and found them to be effective in regenerating partial- and full-thickness cartilage lesions and in improving ICRS scores [99,100,101,102].

Of the studied animal models, since the anatomy and biomechanics of the goat knee joint are the closest to those of humans, and the thickness of its cartilage is adequate, goat is considered to be the most ideal model for cartilage defect healing in large animals.

## 6. Clinical Trials

In addition to animal experiments, MSCs have also shown good progress in clinical trials (Table 2).

### 6.1. OA Treatment with BM-MSCs

#### 6.1.1. Autologous MSCs Generated from Bone Marrow

Human OA knee treatment using MSCs has moved into the clinical research phase, based on promising in vitro and preclinical outcomes [119]. Several groups that administered a single injection of BM-MSCs found clinical improvements [63,64,75,104]. Wakitani et al. [103] found that cartilage abnormalities in the knees of patients with OA might be cured by using MSCs produced from the patient’s own bone marrow. It has been certified that BM-MSCs exhibit a higher histological grade under arthroscopy, indicating that MSCs repair and rebuild cartilage through immunomodulation and can alleviate the symptoms of arthritis. Reports of considerable cartilage and meniscus development after injecting BM-MSCs isolated from the patient’s own tissue to treat joint pain and improve mobility have been encouraging, as described by Centeno et al. [104].

In addition, twelve individuals with OA were treated with intra-articular injections of autologous BM-MSCs, proving the viability and safety of this technique. During the 2 years of treatments, no serious side effects were observed and claims of reduced pain and enhanced cartilage quality were verified [69,105].

In a clinical trial conducted by Lamo-Espinosa et al. [106], 30 patients were randomly divided into two groups: hyaluronic acid was injected into the knees of one group, and the other group received injections of hyaluronic acid containing BM-MSCs. Stochastically, the group containing BM-MSCs was divided into two subgroups: BM-MSCs (low concentrations) + hyaluronic acid and BM-MSCs (high concentrations) + hyaluronic acid. The experimental results suggested that no obvious adverse reactions were found. The pain, OA index, and joint mobility were dramatically optimized in the BM-MSC group, with a high concentration of BM-MSCs leading to greater benefits. Six months later, pain was significantly alleviated and the patients’ activity levels increased. Furthermore, the histology of a specimen taken from one patient one year later revealed the formation of fibrocartilage. Four years later, the same group reported on the utilization of autologous platelet-rich plasma (PRGF^®^) as an adjuvant of MSCs in a phase II randomized controlled clinical trial, which resulted in improved visual analogue scale (VAS) and Western Ontario and McMaster Universities Osteoarthritis Index (WOMAC) scores after follow-up, suggesting that the two are viable treatment options for knee osteoarthritis [107].

Moreover, Lamo-Espinosa et al., further investigated the outcomes of the two pilot studies mentioned above using a Huskisson map to facilitate the quantification and comparability of therapeutic effects. The investigation revealed that when knee joints were treated with varying doses of autologous cells (1, 4, and 100 million), similar healing responses occurred and the effect could last for years [120].

#### 6.1.2. Allogeneic MSCs Generated from Bone Marrow

In a randomized, double-blind controlled trial, allogeneic BM-MSCs were administered 7–10 days after meniscectomy. After 12 months, meniscal volumes (24% of patients in the group injected with 50 × 10^6^ BM-MSCs and 6% in the group injected with 150 × 10^6^ BM-MSCs) had considerably increased, as quantitative MRI measurements and pain levels were significantly lower in the treatment group than in the placebo group [108]. Additionally, a recent study on allogeneic BM-MSC injection confirmed its safety [109]. In a clinical investigation using intra-articular allogeneic BM-MSCs, 60 OA patients were randomly split into groups to obtain varying dosages of cell treatment (ranging from 25 to 150 million cells) or a placebo. The subjective parameters (VAS, ICOAP, and WOMAC-OA scores) of the 25-million-cell dose group tended to be ameliorated. After 12 months, despite the difference from the placebo group not being statistically significant, high-dose patients were more likely to face adverse effects (50, 75, and 150 million cells). The most prevalent side effects typically included knee discomfort and edema [110].

### 6.2. OA Treatment with AD-MSCs

A variety of concentrations of AD-MSCs were intra-articularly injected by Jo et al., to evaluate their effectiveness [65]. High doses of MSCs emerged as a promising prospect during the treatment of knee OA (KOA), including the regeneration of hyaline articular cartilage, a reduction in cartilage defects, the amelioration of knee pain and function, and the absence of adverse effects at the 10 × 10^7^ dose. AD-MSCs were percutaneously injected in combination with arthroscopic debridement in a phase III case–control study including 25 patients with KOA. Pain was reduced and knee function was rapidly improved without risk to the patients [111]. Pers et al. [112] reported that autologous AD-MSCs were used to treat 18 patients with KOA, with the groups receiving either a low dosage (2 × 10^6^ cells), a medium dose (10 × 10^6^ cells), or a high dose (50 × 10^6^ cells). The findings demonstrated that the autologous AD-MSCs were safe and the early clinical outcomes were successful, with even the low-dosage group considerably outperforming the baseline index. Freitag et al. [113] presented a randomized controlled trial and demonstrated potential for significant improvements in clinical pain and AD-MSC functions. In the experiments conducted by Li et al. [114], significant improvements in WOMAC and VAS scores were seen. Lu et al., studied AD-MSCs of autologous and allogeneic origin, both of which played a large role in improving arthritis pain and function [115,116].

### 6.3. OA Treatment with HUC-MSCs

Through dry arthroscopy, Sadlik et al. [121,122] implanted HUC-MSCs covered with collagen scaffolds into the cartilage defects of five patients. Infection, excessive synovial hyperplasia, tumor formation, transplantation rejection, and graft-versus-host reactions were not observed following surgery. Knee joint discomfort was relieved in all instances in the early stages, though the long-term effects still need to be further evaluated. Furthermore, patients with moderate-to-severe OA were treated with HUC-MSCs by Wang et al. [117]. The cell treatment group showed considerable improvements in the SF-36 scale, Lysholm, and WOMAC scores after 3 and 6 months. In addition, it was shown that placental MSCs may have use in sports medicine [123].

### 6.4. OA Treatment with HUCB-MSCs

**Figure 3:**
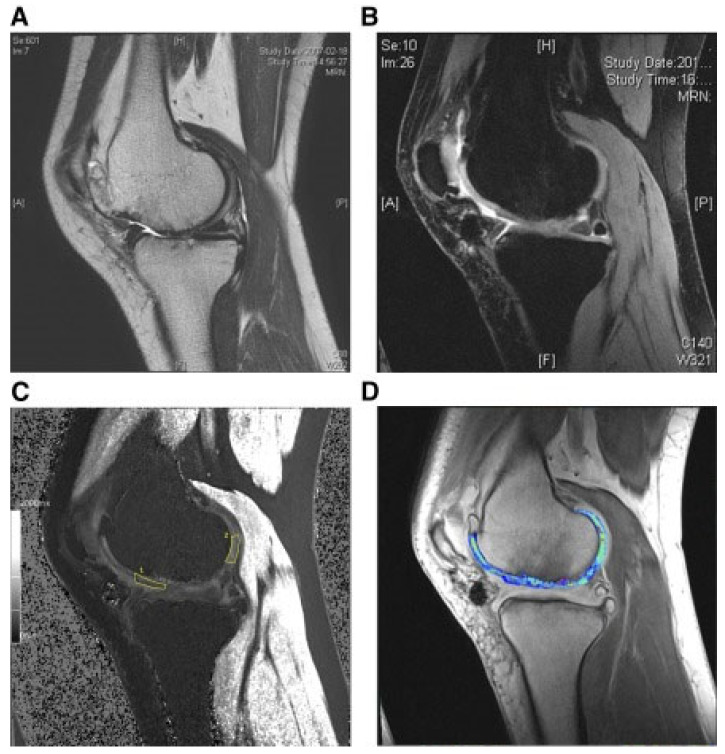
MRI evaluation of cartilage regeneration 3 years after HUCB-MSC treatment. (**A**) A preoperative cartilage defect. (**B**) Cartilage regeneration at 3 years post-transplantation. (**C**) The change in relative cartilage relaxation rate was calculated by sampling in the marked area. (**D**) An increased GAG content is shown in relation to the blue signal. Reprinted from [118]. MRI, magnetic resonance imaging; HUCB-MSCs, human umbilical cord blood-derived mesenchymal stem cells; GAG: glycosaminoglycan.

The safety and effectiveness of human umbilical cord blood-derived MSCs (HUCB-MSCs) for treating cartilage regeneration were also evaluated. HUCB-MSCs were used to treat seven patients with KL grade III OA and grade Ⅳ cartilage defects according to the International Association for Cartilage Repair. Allogeneic HUCB-MSCs were grown in vitro and then combined with a HA hydrogel and applied to the injury site. A microfracture protocol was used in conjunction with these cells. After 12 weeks, the repaired tissue seemed mature, and after 24 weeks, the clinical score increased. The stability of the clinical improvement throughout the course of the 7-year follow-up was also noteworthy. Histology was used to reveal hyaline cartilage one year after surgery while MRI was used to reveal cartilage regeneration 3 years after surgery, as seen in Figure 3 [118] (for which gadolinium-DTPA was used as the contrast-agent visualized with the blue color). During the 7-year follow-up period, no cases of malignancies were found and only five patients appeared to possess moderate-to-severe treatment-related adverse events.

## 7. Meta-Analysis

The safety and effectiveness of MSC transplantation used to treat OA have been demonstrated by multiple meta-analyses. For example, Peeters et al. [124] performed a systematic analysis of autologous MSCs in the treatment of OA, including 844 patients who were followed for an average of 21 months. In all included studies, intra-articular injections of MSCs from various sources (bone marrow, fat, and umbilical cord blood) were confirmed to be effective and safe in the treatment of osteoarthritis. Four individuals experienced severe problems, including the infection of the bone marrow and pulmonary embolism (thought to be connected to bone marrow extraction), and 22 probable adverse effects, mostly characterized by increased joint pain and edema, were associated with the procedure. The clinical effectiveness and safety of MSCs from various sources for the treatment of KOA were evaluated in a meta-analysis including 18 trials and 565 patients [125], which indicated that the utilization of MSCs may enhance the fundamental assessment, physical performance, pain alleviation, and safety of patients with KOA.

In another meta-analysis, 11 clinical trials involving 582 KOA patients were included. After 24 months of therapy, the VAS scores of a group injected with MSC from diverse sources dramatically were reduced (bone marrow, fat, etc.) relative to the control group, but the IKDC scores significantly grew. After 12 months of follow-up, the WOMAC and Lequesne scores of the MSC treatment group were also dramatically reduced. An examination of Lysholm and Tegner scores also revealed that the therapeutic efficacy of the MSCs was superior. However, Xing et al. [126] reported that of the four systematic reviews of MSCs in the treatment of OA, only one had a moderate potential for bias [127] while the others had a considerable risk of bias [125,128,129]. Although the authors were somewhat confident in the safety of BM-MSCs for treating KOA, they were far less confident in their effectiveness due to the limitations of the available data [126].

There were 79 randomized controlled trials included in a recent meta-analysis. Autologous conditioned serum, bone marrow aspirate concentrate, botulinum toxin, corticosteroids, hyaluronic acid, MSCs, ozone, saline placebos, platelet-rich plasma, plasma high in growth factor, and stromal vascular fraction were all tested as intra-articular injectables (SVF). The results indicated that MSC treatment was not recommended for OA [130]. Another recent meta-analysis only included papers that fulfilled the inclusion criterion eighteen times. This meta-analysis established the safety and effectiveness of MSC treatment for KOA in older persons, showing that these therapies decrease pain and enhance knee function in symptomatic KOA.

## 8. Conclusions and Perspectives

OA is a complex disease that is steadily affecting a greater number of people as the population ages. Currently, the treatment of OA is focused on relieving clinical symptoms, improving joint mobility, and enhancing the quality of life of patients. However, the process of articular cartilage degeneration has not been reversed, so a complete cure for OA cannot be achieved. With increasing research into cell regeneration and cartilage repair, the advantages of a wide source of single-nucleated cells and mesenchymal stem cells and their ability to proliferate are expected to slow down the progression of osteoarthritis and result in a complete cure. Preclinical studies and clinical trials have highlighted the therapeutic potential of MSCs in the treatment of OA. However, while MSCs have therapeutic value, there is still a considerably long way to explore until they can be clinically applied. (1) Based on the current information, it is premature to draw conclusions about the ultimate effectiveness of MSCs. There is a need for additional radiological and histological data, necessitating additional clinical trials to generate additional data that can be used to provide future recommendations for treating OA. (2) Therapeutic effectiveness varies based on the source of MSCs. This merits further research to determine which source is the optimal treatment for OA under different conditions. (3) The potential therapeutic mechanisms of mesenchymal stem cells for OA require further investigation. We think that the advances in MSC-based approaches to OA treatment, as well as the further discovery of mechanisms, will provide us with an unparalleled chance of improving the treatment of OA. (4) Injecting MSCs has significant limitations, including cell death at injection and significant leakage at the injection site. Overcoming these limitations using a combination of biomaterials and stem cells is essential for the treatment of OA. In other words, biomaterial-assisted cell cartilage repair may be the main direction and source of hope for the future treatment of osteoarthritis.

## Figures and Tables

**Table 1:** Overview of preclinical studies applying different sources of MSCs for OA treatment.

References	Year	Animal Model	Source of MSCs	Lesion Preparation	Outcomes
Horie et al. [76]	2009	Rat	Autologous synovium	Meniscectomy	MSCs clung to meniscus lesions and directly developed into meniscal cells to facilitate meniscus repair and regeneration.
Horie et al. [77]	2012	Rat	Xenogeneic (human) bone marrow	Hemi-meniscectomy	Type II collagen expression levels rose and the progression of OA was dramatically slowed.
Cui et al. [78]	2015	Rat	Allogeneic bone marrow	ACLT/medial meniscus excising	The Mankin score was significantly improved, and the mRNA expression of type II collagen increased.
van Buul et al. [79]	2014	Rat	Allogeneic bone marrow	Inducted by MIA injection	The local injection of MSCs significantly improved joint function, but there was no statistical difference in cartilage improvement, subchondral bone pathology, and synovitis.
Ozeki et al. [61]	2016	Rat	Xenogeneic (human) synovium	ACLT	Injected Sy-MSCs increased the expression of genes associated with chondroprotection such as PRG-4 and BMP-2 by more than 50 fold.
He et al. [80]	2020	Rat	Allogeneic bone marrow	Inducted by sodium iodoacetate injection	The COL2A1 protein was significantly upregulated and the MMP13 protein was significantly downregulated in cartilage tissue after exosome therapy.
Xing et al. [81]	2021	Rat	Xenogeneic (human) embryonic stem cell	ACLT	The better therapeutic benefits of many injections of embryonic MSCs were maintained in both the short- and long-term after treatment.
Yang et al. [82]	2022	Rat	Autologous adipose tissue	Inducted by sodium iodoacetate injection	Treatments using adipose-derived stem cells aided articular cartilage repair.
Zellner et al. [68]	2017	Rabbit	Autologous and xenogeneic (human) bone marrow	Punch defects on the lateral meniscus	The human MSCs demonstrated the considerably increased expression of the collagen type II gene and the synthesis of collagen.
Mata et al. [83]	2017	Rabbit	Xenogeneic (human) dental pulp	Defects in femoral trochlear groove	Obvious cartilage regeneration was observed 3 months after operation.
Riester et al. [84]	2017	Rabbit	Xenogeneic (human) adipose tissue	Bilateral medial anterior hemi-meniscectomy	The tolerance was good, and no evidence of intra-articular joint tissue damage was found.
Jeon et al. [85]	2020	Rabbit	Xenogeneic (human) umbilical cord blood	ACLT	Rabbit synovial fluid and joints treated with HUCB-MSCs showed reduced inflammation and improved proteoglycan and collagen type 2 production and structure.
Pei et al. [86]	2009	Rabbit	Allogeneic synovium	Full-thickness femoral condyle cartilage defects	The regenerated cartilage appeared as smooth hyaline cartilage at a 6-month follow-up.
Lee et al. [87]	2013	Rabbit	Autologous synovium	Osteochondral defects on trochlear groove of femur	The treated group showed significantly improved microscopic and macroscopic scores at a 6-month follow-up.
Shimomura et al. [88]	2014	Rabbit	Allogeneic synovium	Osteochondral defects on the femoral groove	Subjects using Sy-MSCs and HA exhibited faster integration and the improved appearance of osteochondral bone compared with controls using only HA.
Li et al. [89]	2016	Rabbit	Autologous synovium	Osteochondral defects on right knee trochlea	The treated animals had a higher quality of tissue.
Schmal et al. [90]	2018	Rabbit	Allogeneic synovium	Osteochondral defects on medial femoral condyle	Macroscopic regenerative capacity increased.
Murphy et al. [91]	2003	Goat	Autologous bone marrow	Surgical removal of the medial meniscus and anterior cruciate ligament reconstruction	Cartilage tissue regeneration was observed, but a relative lack of labeled MSCs was found in the regenerated cartilage area.
Saw et al. [92]	2009	Goat	Autologous bone marrow	Arthroscopic subchondral drilling	Tissue integration and tissue repair could be improved with the use of a bone marrow aspiration primer combined with hyaluronic acid.
Feng et al. [93]	2018	Goat	Allogeneic adipose tissue	Anterior cruciate ligament resection/medial meniscectomy	An examination using MRI, macroscopic and microcomputer tomography, and cartilage-specific staining showed that the AD-MSCs + HA treatment group retained the typical characteristics of articular cartilage, effectively blocked the progress of OA, and boosted cartilage regeneration.
McIlwraith et al. [94]	2011	Horse	Allogeneic bone marrow	Subchondral bone microfracture	The histological analysis of the intra-articular BM-MSC injection group revealed improved proteoglycan and tissue stiffness in the restored cartilage.
Black et al. [95]	2007	Dog	Autologous adipose tissue	Functional disabilities	The claudication index, pain score, and range of motion were substantially increased.
Black et al. [96]	2008	Dog	Autologous adipose tissue	Functional disabilities	The claudication and range of movement of dogs were significantly improved.
Huňáková et al. [97]	2020	Dog	Allogeneic adipose tissue	Untreated elbow dysplasia	The double intra-articular administration of canine adipose tissue derived from Labrador retrievers improved the functional ability of dogs.
LEE et al. [98]	2007	Pig	Autologous bone marrow	Cartilage defect in themedial femoral condyle	Cartilage defect cartilage healing improved.

MSCs: mesenchymal stem cells; OA: osteoarthritis; ACLT: anterior cruciate ligament transection; MIA: mono-iodoacetate; Sy-MSCs: synovium-derived mesenchymal stem cells; HUCB-MSCs: human umbilical cord blood-derived mesenchymal stem cells; HA: hyaluronic acid; MRI: magnetic resonance imaging; AD-MSCs: adipose tissue-derived mesenchymal stem cells; BM-MSCs: bone marrow-derived mesenchymal stem cells.

**Table 2:** Overview of clinical studies applying different sources of MSCs for OA treatment.

References	Years	Condition	Sample Size	Source of MSCs	Mode of Administration	Follow-Up	Outcomes
Wakitani et al. [103]	2007	Full-thickness articular cartilage defects of the patellofemoral joints	N = 3; Females = 1; Males = 2; Mean age = 40 years	Autologous BM-MSCs	Surgical implantation in the form of collagen gel wrapped around BM-MSCs (5 × 10^6^ cells/mL)	1 year	IKDC scores all improved to more than 60. MRI showed defects that were repaired with the fibrocartilaginous tissue.
Centeno et al. [104]	2008	Degenerative knee osteoarthritis	N = 1; Females = 0; Males = 1; Mean age = 46 years	Autologous BM-MSCs	Percutaneously injection (2.24 × 10^8^)	6 months	After 3 months, the VAS dropped from 4 to 0.38, a 95% reduction. The joint range of motion increased from −2 degrees to +3 degrees when stretching.
Orozco et al. [69]	2013	K–L grade II–IV knee osteoarthritis	N = 12; Females = 6; Males = 6; Mean age = 49 ± 5 years	Autologous BM-MSCs	Intra-articular injection (40 × 10 cells)	1 year	Quantification of cartilage quality by T2relaxation measurements demonstrated a 27% decrease in poor cartilage areas on average; the mean VASvalues of 45 and 47 were recorded.
Orozco et al. [105]	2014	K–L grade II–IV knee osteoarthritis	N = 12; Females = 6; Males = 6; Mean age = 49 ± 5 years	Autologous BM-MSCs	Intra-articular injection (40 × 10 cells)	2 years	The therapeutic efficiency was 0.71 for VAS and 0.66 for the Lequesne severity index; the WOMAC score varied between 0.44 and 0.78.
Lamo-Espinosa et al. [106]	2016	K–L grade II–IV knee osteoarthritis	N = 30; Females = 11; Males = 19; Mean age = 61 years	Autologous BM-MSCs	Intra-articular administration (10 or 100 × 10^6^)	1 year	The median VAS scores in the control, low-dose, and high-dose groups changed from 5, 7, and 6 to 4, 2, and 2, respectively, after 1 year. The WOMAC scores in the high-dose group showed a 16.5-point improvement after 1 year.
Lamo-Espinosa et al. [107]	2020	K–L grade II–IV knee osteoarthritis	N = 60; Females = 27; Males = 3; Mean age = 55 years	Autologous BM-MSCs	Lateral patellar administration (100 × 10^6^)	1 year	The mean VAS values in the PRGF^®^ and BM-MSC with PRGF^®^ groups changed from 5 and 5.3 to 4.5 and 3.5, respectively, after 1 year. The WOMAC scores changed from 31.9 and 33.4 to 22.3 and 23.0, respectively.
Vangsness et al. [108]	2014	After partial meniscectomy	N = 55; Females = 20; Males = 35; Mean age = 46 years	Allogeneic BM-MSCs	Superolateral knee injection (50 or 150 × 10^6^)	2 years	Meniscal volumes (24% of patients in the group injected with 50 × 10^6^ BM-MSCs and 6% in group injected with 150 × 106 BM-MSCs) considerably increased.
Vega et al. [109]	2015	K–L grade II–IV chronic knee osteoarthritis	N = 30; Females = 17; Males = 13; Mean age = 57 ± 9 years	Allogeneic BM-MSCs	Intra-articular injection (40 × 10^6^)	1 year	The mean VAS scores in the experimental group and the control group increased from 54 and 64 to 33 and 51, respectively. The WOMAC pain scores increased from 46 and 50 to 30 and 44, respectively.
Gupta et al. [110]	2016	K–L grade II–III knee osteoarthritis	N = 60; Females = 45; Males = 15; Mean age = 56 ± 7.43 years	Allogeneic BM-MSCs	Intra-articular injection (25, 50, 75, or 150 × 10^6^)	1 year	The WOMAC and total ICOAP scores decreased in all treatment groups, the VAS score decreased in all but the 150 × 10^6^ group, and the 25 × 10^6^ group had the largest decreases (64.8%, 34.6%, and 67.4%).
Jo et al. [65]	2014	K–L grade III–IV knee osteoarthritis	N = 18; Females = 15; Males = 3; Mean age = 62 years	Autologous AD-MSCs	Intra-articular injection (1, 5, or 10 × 10^7^)	6 months	The WOMAC score in the 10 × 10^7^ group decreased by 39%, and the knee score of KSS in the 1 and 10 × 10^7^ groups increased by 91% and 50%, respectively.
Koh et al. [111]	2012	K–L grade II–IV knee osteoarthritis	N = 25; Females = 17; Males = 8; Mean age = 54.1 years	Autologous AD-MSCs	Percutaneous injection combined with arthroscopic debridement (1.89 × 10^6^)	16.4 months	The mean Lysholm and Tegner activity scales in the studygroup improved by 26.9 and 1.3 points, respectively; the VAS score decreased by 2.2 points.
Pers et al. [112]	2016	K–L grade III–IV knee osteoarthritis	N = 18; Females = 10; Males = 8; Mean age = 64.6 years	Autologous AD-MSCs	Intra-articular injection (2, 10, or 50 × 10^6^)	6 months	No serious adverse events were reported, and the WOMACpain score decreased by 30.7 ± 10.7 mm in the 2 × 10^6^ group.
Freitag et al. [113]	2019	K–L grade II–III knee osteoarthritis	N = 30; Females = 14; Males = 16; Mean age = 53.6 years	Autologous AD-MSCs	Intra-articular injection (100 × 10^6^)	1 year	NPRS was improved by 69% in the treatment group. The mean WOMAC score changed from 57 to 85.7.
Lee et al. [114]	2019	K–L grade II–IV knee osteoarthritis	N = 24; Females = 18; Males = 6; Mean age = 62.7 years	Autologous AD-MSCs	Intra-articular injection (1 × 10^8^)	6 months	The WOMAC and VAS scores in the AD-MSC group changed from 60 and 6.8 to 26.7 and 3.4, respectively.
Lu et al. [115]	2019	K–L grade I–III knee osteoarthritis	N = 52; Females = 46; Males = 6; Mean age = 55 years	Autologous AD-MSCs	Intra-articular injection (5 × 10^7^)	1 year	The total volume of articular cartilage in the treatment group increased by 193.36 ± 282.80 mm^3^ compared with the baseline for the left knee and 108.70 ± 220.13 mm^3^ for the right knee in 1 year.
Lu et al. [116]	2020	K–L grade II–III knee osteoarthritis	N = 22; Females = 19; Males = 3; Mean age = 57.93 years	Allogeneic AD-MSCs	Intra-articular injection (1, 2, or 5 × 10^7^)	48 weeks	A joint assessment of VAS, SF-36, and WOMAC scores improved, with averages of 2.03, 15.3, and 16.97, respectively, in three experimental groups.
Wang et al. [117]	2016	Moderate or severe degenerative knee osteoarthritis	N = 36; Females = 15; Males = 21; Mean age = 53.33 years	Allogeneic HUC-MSCs	Intra-articular injection ((2–3) × 10^7^)	6 months	The Lysholm and WOMAC at 1–6 months and the SF-36 scale score at 2–6 months were significantly better than before treatment in the cell treatment group.
Park et al. [118]	2017	K–L grade III knee osteoarthritis and ICRS grade IV lesions	N = 7; Females = 5; Males = 2; Mean age = 58.7 years	Allogeneic HUCB-MSCs	Surgical implantation of a complex containing stem cells and hyaluronic acid hydrogel (0.5 × 10^7^)	7 years	Maturing repair tissue was observed at the 12-week arthroscopic examination. The 100 mm VAS and IKDC scores changed from 49.1 and 39.1 to 19.3 and 63.2, respectively, at 24 weeks.

IKDC: the international knee documentation committee knee evaluation form; VAS: visual analogue scale; WOMAC: the Western Ontario and McMaster Universities Osteoarthritis Index; ICOAP: intermittent and constant osteoarthritis pain; KSS: American knee society knee score; NPRS: numerical pain rating scale; HUC-MSCs: human umbilical cord-derived mesenchymal stem cells; SF-36: the MOS item short from health survey.

## Data Availability

Not applicable.
